# The value of adjusted PSAD in prostate cancer detection in the Chinese population

**DOI:** 10.3389/fonc.2024.1462997

**Published:** 2024-10-02

**Authors:** Fangming Wang, Meng Fu, Yuzhe Tang, Jianxing Li

**Affiliations:** Department of Urology, Tsinghua University Affiliated Beijing Tsinghua Changgung Hospital, Tsinghua University Clinical Institute, Beijing, China

**Keywords:** prostate cancer (PCa), biopsy, diagnosis, prostate-specific antigen density (PSAD), benign prostatic hyperplasia

## Abstract

**Objective:**

To investigate the value of adjusted prostate-specific antigen density (PSAD^adj^) in the diagnosis of prostate cancer (PCa).

**Methods:**

Data from 410 patients who underwent transrectal ultrasound-guided prostate biopsy were retrospectively analyzed in Beijing Tsinghua Changgung Hospital between November 2014 and March 2024. All patients were divided into PCa and benign prostatic hyperplasia (BPH) groups according to pathological results. Multivariate logistic regression analyses were performed to evaluate the odd ratios (ORs) of predictors for PCa occurrence. Receiver operating characteristic curves were plotted, and the area under the curve (AUC) values were used to assess and compare the diagnostic accuracies of total PSA (tPSA), free-to-total (f/t) PSA, free PSA (fPSA), PSAD, and PSAD^adj^ (PSAD×weight).

**Results:**

There were 166 patients in the PCa group and 244 in the BPH group. Multivariate analyses demonstrated that PSAD was positively correlated with the presence of PCa, with the highest OR value among all PSA-related parameters (OR = 19.075, *p*<0.001). tPSA, fPSAD, PSAD, and PSAD^adj^ had high accuracy in predicting PCa, with AUC values of 0.633, 0.730, 0.778, and 0.780. Of note, PSAD^adj^ had the highest AUC with a sensitivity of 63.3% and specificity of 81.6%. Similarly, in patients with a PSA level in the gray zone, the diagnostic accuracy of PSAD^adj^ in predicting PCa (AUC, 0.709; 95% CI, 0.616–0.802) remained better than other PSA-related markers.

**Conclusion:**

PSAD^adj^ has an advantage over other PSA-related markers in detecting PCa and could be used for making biopsy decisions.

## Introduction

Prostate cancer (PCa) is the world’s second-most frequent cancer and the fifth leading cause of cancer death among men in 2022 (1). Differences in serum prostate-specific antigen (PSA) screening policy at the national level could explain much of the variations in PCa incidence worldwide. The use of PSA as a serum marker has revolutionized PCa diagnosis. However, PSA is organ but not cancer specific; elevated PSA could be observed in benign prostatic hyperplasia (BPH), prostatitis, and other non-malignant conditions (2); and there are no agreed standards for defining PSA thresholds. Therefore, many studies have focused on PSA-derived indicators, such as the free/total PSA (f/tPSA) ratio, free PSA density (fPSAD), and PSA density (PSAD) (3–6), aiming to find the parameter with an ideal diagnostic accuracy. Of note, PSAD, one of the strongest predictors in risk calculators, has received increasing attention in recent years (5, 7–9). The higher the PSAD, the more likely it is that the PCa is detected. Several studies have shown that PSAD has a higher diagnostic value for PCa than PSA alone (10, 11). Moreover, PSAD has been used in addition to multiparametric magnetic resonance imaging (mpMRI) findings to more accurately predict biopsy outcomes and avoid unnecessary biopsies (11–14). PSAD is the level of serum PSA concentration divided by the prostate volume, and it is used to compensate for BPH and prostate size, with densities greater than 0.10–0.15 more suggestive of PCa (5). However, the utility of this method as a screen is limited because of variations in blood volume. For example, the PSAD level measured after water intake or in patients with water retention due to renal insufficiency would underestimate the risk of PCa occurrence. In addition, the obese may be more likely to harbor PCa than the non-obese with the same PSAD value. Therefore, we proposed the concept of adjusted PSAD (PSAD^adj^) (calculated by PSAD×weight) to solve this problem based on the fact that blood volume is proportional to body weight.

Most studies of PSAD parameters were conducted in Western countries; the levels of PSA and prostate size in the Chinese population were different from those in Western countries. More importantly, until now, no information is available on the value of PSAD^adj^ in predicting PCa worldwide. Therefore, in this study, we comprehensively analyzed the diagnostic performance of PSAD^adj^, PSAD, and other PSA-derived parameters among 410 Chinese patients from our center cohort, thereby providing further support for the clinical diagnosis of PCa.

## Patients and methods

### Patient data collection

The current study included patients who underwent an ultrasound-guided transrectal biopsy at Beijing Tsinghua Changgung Hospital between November 2014 and March 2024. All clinicopathological data were collected from the hospital information system. The data included age, body mass index (BMI), tPSA, fPSA, MRI findings, and biopsy pathological results including positive cores and the International Society of Urological Pathology (ISUP) grade. The exclusion criteria of the study were as follows: (1) the presence of a previous prostate biopsy; (2) a failure to estimate prostate volume due to a lack of MRI examination; (3) a PSA level >100ng/ml; (4) acute prostatitis, prostatic massage, digital rectal examination (DRE), or cystoscopy within 2 weeks before the PSA test; and (5) surgical treatments for BPH. For patients treated with a 5α-reductase inhibitor, we recorded the highest PSA and volume before they took medicine. Finally, 410 cases were included.

### Calculation of PSA-derivative parameters

Prostate gland volume was evaluated through the measurement of prostate dimensions obtained from the three-dimensional T2-weighted imaging measurements of mpMRI (transverse diameter × anteroposterior diameter × craniocaudal diameter × 0.52). The ratio of free PSA to total PSA was labeled as f/t PSA. The PSAD was calculated by dividing the serum tPSA by the prostate volume. Similarly, the fPSAD was calculated by dividing the serum fPSA by the prostate volume. PSAD^adj^ was calculated by PSAD multiplied by body weight.

### Prostate biopsy

The prostate biopsy indication in our center followed the guidelines of the Chinese Urology Association: (1) suspicious prostate nodules were found by DRE; (2) suspicious lesions were detected by transrectal ultrasound (TRUS) or MRI; (3) PSA >10 ng/ml, regardless of the value of f/tPSA and PSAD; and (4) PSA=4–10 ng/ml, abnormal f/tPSA or abnormal PSAD value. All patients routinely underwent 3-Tesla MRI examination before biopsy. The MRI inquisition protocol included T1-weighted imaging (T1WI), T2-weighted imaging (T2WI), diffusion weighted imaging (DWI) and dynamic contrast-enhanced MR imaging (DCE-MRI). mpMRI studies were assessed using the Prostate Imaging and Reporting Data System (PI-RADS) version 2.0 (before 2019) and 2.1 (2019 or later) and interpreted by specialized genitourinary radiologists at our center. According to both versions, MRI was considered negative with a score ≤2 and positive with a score ≥3. We performed mpMRI cognitive fusion plus ultrasound-guided transrectal biopsy (using Philips ultrasound, HD15). Specifically, we carried out a systematic biopsy by obtaining 12 systematic cores (the inner and outer sides of the left and right prostatic apex, body, and fundus) and up to 1–2 cores targeting hypoechoic lesions based on real-time ultrasound findings. Finally, the harvested specimen was marked, fixed in strips, and sent to the pathology department for diagnosis.

### Statistical analysis

Data were expressed as means ± SD or median with interquartile ranges for continuous variables and numbers (percentage) for categorical variables. Unpaired t-tests or Mann–Whitney U tests were used to compare the differences between continuous variables. χ2-tests were used to compare the proportions between categorical variables. All included men were divided into two groups according to the pathological result of the prostate biopsy, PCa and BPH groups, and clinical variables including age, BMI, prostate volume, positive MRI percentage, and PSA-derived parameters were compared between the two groups. Multivariate logistic regression analysis was applied to evaluate the prediction efficacy of each PSA-derived parameter (tPSA, f/t PSA, PSAD, fPSAD, PSAD^adj^) for predicting PCa in four different models: Model 1 corrected for covariates including age, BMI, volume, and positive MRI; Model 2 corrected for age, BMI, volume, and positive MRI; Model 3 corrected for age, BMI, and positive MRI; and Model 4 corrected for age, PSAD^adj^, and positive MRI. Receiver operating characteristic (ROC) curves were plotted and the area under the ROC curve (AUC) was calculated to compare the predictive values of PSA-derived parameters on PCa detection. The cutoff value for each PSA-derived parameter was determined from the ROC curves, and the sensitivity and specificity for the prediction of PCa were calculated for the overall study population and those with PSA ranging from 4.0 ng/ml to 10.0 ng/ml. The Youden index was used to identify the optimal cutoff point (Youden index=sensitivity+specificity-1). All tests were two-sided and *p*<0.05 was considered significant. The statistical analyses were performed with SPSS version 22.0 software (SPSS Inc., Chicago, IL, USA) and GraphPad Prism 8 software.

## Results

### Comparison of clinicopathological parameters between the PCa and BPH groups

**Figure 1:**
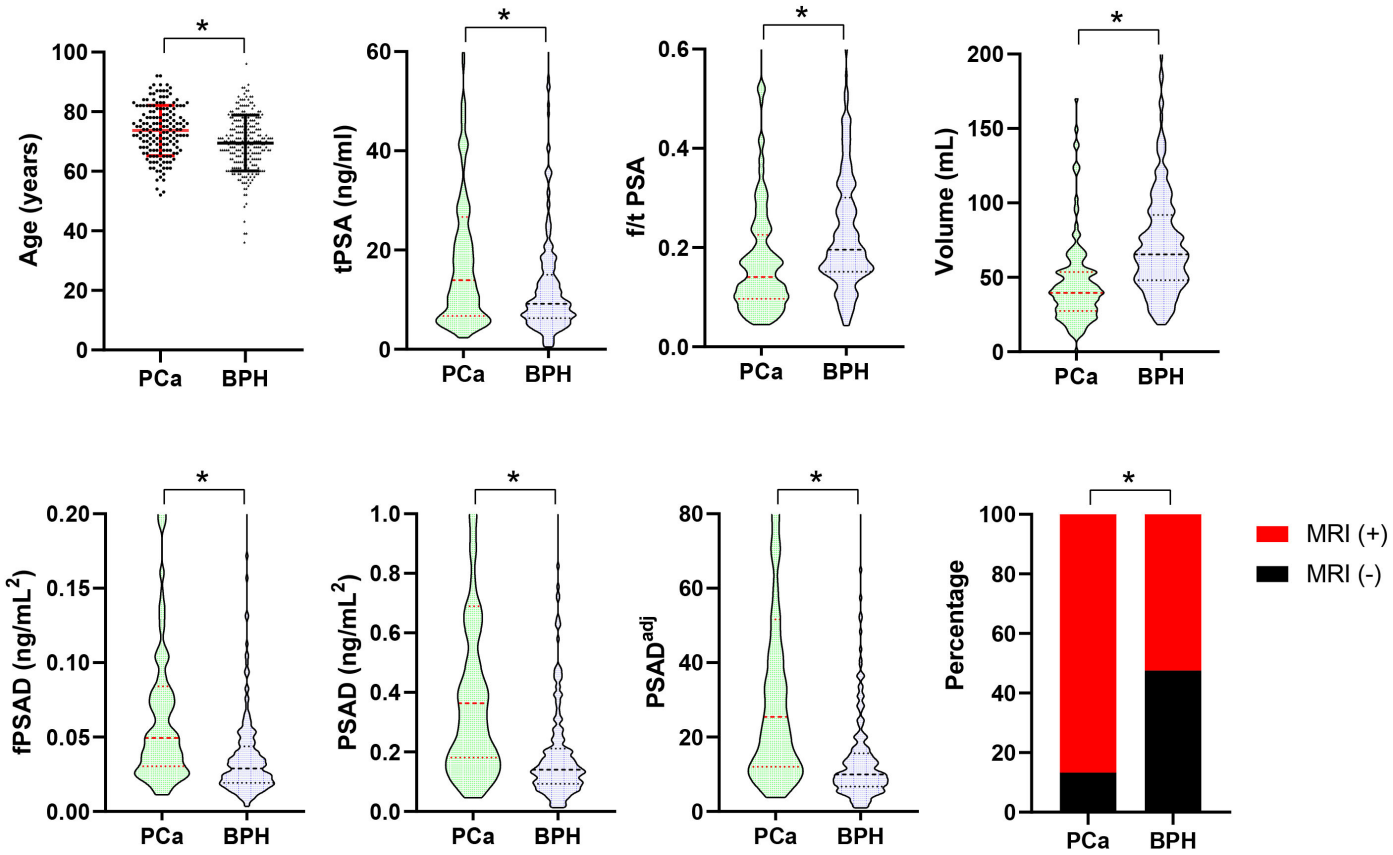
Comparison of age, PSA-derived parameters, prostate volume, and positive MRI percentages between the PCa (n=166) and BPH (n=244) groups. Data are expressed as mean ± SD, median (interquartile range), or a percentage. The median with interquartile range is shown in a violin plot (the dotted line). Statistical significance was determined using an unpaired t-test, the Mann–Whitney U test, or a χ2-test. *p<0.05. PCa, prostate cancer; BPH, benign prostatic hyperplasia; PSA, prostate-specific antigen; tPSA, total PSA; f/t PSA, free-to-total PSA ratio; fPSAD, free prostate-specific antigen density; PSAD^adj^, adjusted PSAD; MRI, magnetic resonance imaging.

The clinicopathological characteristics, including age, BMI, volume, PSA-derivative parameters, and MRI findings, of the PCa and BPH groups are shown in **Table 1**. Among the 410 men biopsied, 166 were diagnosed with PCa, resulting in an overall positive biopsy rate of 40.5% (PCa group, n=166; BPH group, n=244). The age, tPSA, fPSAD, PSAD, PSAD^adj^, and positive MRI percentages were significantly higher in the PCa group than in the BPH group (p<0.001), whereas f/t PSA and prostate volume were significantly lower in the PCa group than in the BPH group (*p*<0.001) (**Figure 1**). In PCa groups, the percentages of positive biopsy cores were 13.9%, 8.4%, 10.8%, 8.4%, 7.2%, 8.4%, 9.0%, 6.0%, 6.0%, 1.8%, 3.0%, 4.2%, and 12.7%, respectively, from cores 1 to 13. In addition, the distribution of the ISUP grade was as follows: grade 1 (24.7%, 41/166), grade 2 (11.4%, 19/166), grade 3 (13.3%, 22/166), grade 4 (26.5%, 44/166), and grade 5 (24.1%, 40/166). Collectively, levels of PSA-related parameters and the distribution of MRI findings were significantly different between the PCa and BPH groups.

**Table 1:** Comparison of demographic and clinical characteristics between the PCa and BPH groups.

	PCa (n = 166)	BPH (n = 244)	*p* value
Age (years)	73.7 ± 8.5	69.5 ± 9.4	**<0.001**
BMI (kg/m^2^)	24.9 ± 3.5	24.7 ± 3.4	0.562
tPSA (ng/ml)	13.9 (6.7-26.7)	9.2 (6.3-15.0)	**<0.001**
f/t PSA	0.14 (0.10-0.23)	0.20 (0.15-0.30)	**<0.001**
Volume (ml)	39.5 (27.3-53.6)	65.3 (48.0-91.9)	**<0.001**
fPSAD (ng/ml^2^)	0.049 (0.030-0.084)	0.029 (0.019-0.044)	**<0.001**
PSAD (ng/ml^2^)	0.36 (0.18-0.69)	0.14 (0.09-0.21)	**<0.001**
PSAD^adj^ (ng/ml)	25.4 (12.0-51.6)	10.0 (6.7-15.6)	**<0.001**
Positive MRI [n (%)]	144 (86.7)	128 (52.5)	**<0.001**
Positive cores [n (%)]			
1	23 (13.9)		
2	14 (8.4)		
3	18 (10.8)		
4	14 (8.4)		
5	12 (7.2)		
6	14 (8.4)		
7	15 (9.0)		
8	10 (6.0)		
9	10 (6.0)		
10	3 (1.8)		
11	5 (3.0)		
12	7 (4.2)		
13	21 (12.7)		
Biopsy ISUP grade (n(%))			
1	41 (24.7)		
2	19 (11.4)		
3	22 (13.3)		
4	44 (26.5)		
5	40 (24.1)		

Data are expressed as n (%), mean ± SD, or median (interquartile range). The bold value indicated statistical significance. PCa, prostate cancer; BPH, benign prostatic hyperplasia; BMI, body mass index; tPSA, total prostate-specific antigen; fPSAD, free prostate-specific antigen density; PSAD^adj^, adjusted PSAD; MRI, magnetic resonance imaging; ISUP, international society of urological pathology.

### Correlations of PSA-derivative parameters and positive MRI with the presence of PCa

We performed multivariate logistic regression analysis to evaluate the correlations of PSA-derivative parameters and other clinical variables with PCa occurrence. As shown in **Table 2**, in model 1, we revealed that age, tPSA, and positive MRI were significantly and positively correlated with the presence of PCa (OR=1.055, 95% CI: 1.023–1.088, *p*=0.001; OR=1.046, 95% CI: 1.019–1.073, *p*=0.001; OR=3.156, 95% CI: 1.693–5.883, *p*<0.001), whereas prostate volume was significantly and negatively correlated with the presence of PCa (OR=0.965, 95% CI: 0.954-0.977, *p*<0.001). In model 2, we found that f/tPSA was negatively correlated with the presence of PCa (OR=0.061, 95% CI: 0.006–0.575, *p*=0.015), and the relationship of age, volume, and positive MRI with PCa remained as significant as in model 1. More importantly, in model 3, we demonstrated that age, PSAD, and positive MRI were significantly and positively correlated with PCa occurrence (OR=1.052, 95% CI: 1.021–1.083, *p*=0.001; OR=19.075, 95% CI: 6.677–54.497, *p*<0.001; OR=3.452, 95% CI: 1.917–6.218, *p*<0.001). Moreover, in model 4, we demonstrated that age, PSAD^adj^, and positive MRI were significantly and positively correlated with PCa occurrence (OR=1.053, 95% CI: 1.022–1.084, *p*=0.001; OR=1.043, 95% CI: 1.028–1.059, *p*<0.001; OR=3.921, 95% CI: 2.150–7.151, *p*<0.001). However, the BMI and fPSA results were not associated with PCa (p>0.05 in 3 models; p=0.879). It is noteworthy that the OR value of PSAD was the highest among all PSA-related parameters in the mentioned models, suggesting the great diagnostic value of PSAD in the detection of PCa. Next, we focused on the predictive value of PSAD and PSAD^adj^ by plotting ROC curves.

**Table 2:** Multivariate analysis to compare the independent correlations between different PSA-related parameters and the presence of PCa in biopsied prostate patients.

atient	Variables	Multivariate mode		
		OR	95% CI	*p* value
1	Age	1.055	1.023-1.088	**0.001**
	BMI	1.072	0.994-1.157	0.072
	tPSA	1.046	1.019-1.073	**0.001**
	fPSA	1.011	0.882-1.157	0.879
	Volume	0.965	0.954-0.977	**<0.001**
	Positive MRI	3.156	1.693-5.883	**<0.001**
2	Age	1.060	1.029-1.092	**<0.001**
	BMI	1.059	0.984-1.140	0.128
	f/tPSA	0.061	0.006-0.575	**0.015**
	Volume	0.973	0.963-0.983	**<0.001**
	Positive MRI	3.826	2.078-7.044	**<0.001**
3	Age	1.052	1.021- 1.083	**0.001**
	BMI	1.044	0.973-1.121	0.233
	PSAD	19.075	6.677-54.497	**<0.001**
	Positive MRI	3.452	1.917-6.218	**<0.001**
4	Age	1.053	1.022-1.084	**0.001**
	PSAD^adj^	1.043	1.028-1.059	**<0.001**
	Positive MRI	3.921	2.150-7.151	**<0.001**

Multivariate regression models are shown. The bold value indicated statistical significance. The dependent variable was presence of PCa. PSA, prostate-specific antigen; PCa, prostate cancer; BMI, body mass index; tPSA, total PSA; fPSA, free PSA; MRI, magnetic resonance imaging; f/t PSA, free/total prostate specific antigen ratio; PSAD, prostate-specific antigen density; PSAD^adj^, adjusted PSAD.

### The diagnostic performance of PSAD^adj^ and other PSA-derivative parameters

**Figure 2:**
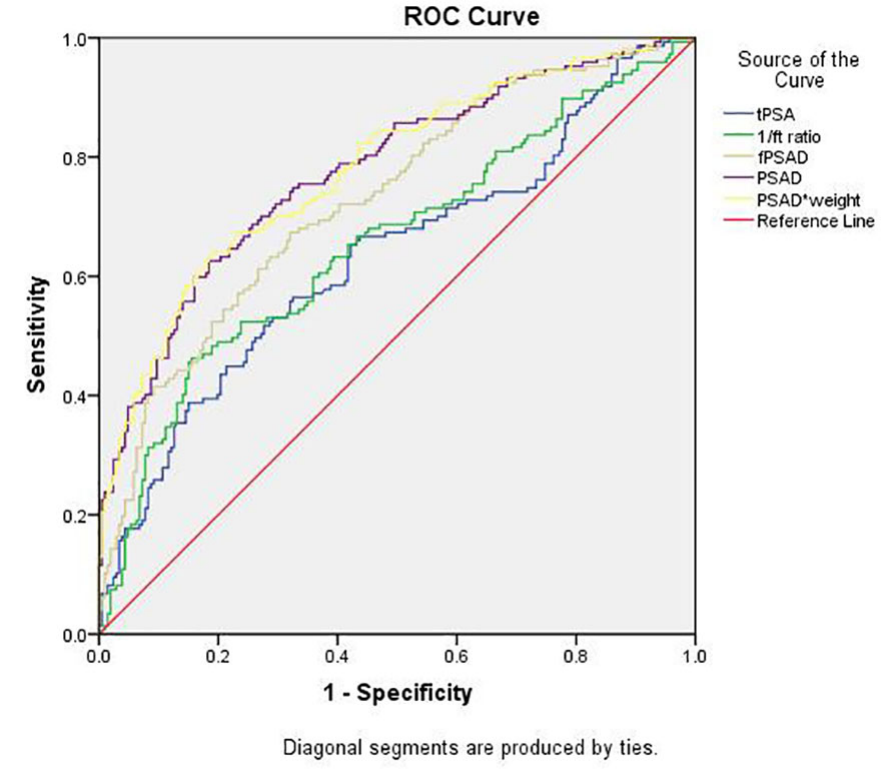
Comparison of the diagnostic efficacy of tPSA, f/t PSA, fPASD, PSAD, and PSAD^adj^ for PCa in whole patients regardless of PSA levels. PCa, prostate cancer; PSA, prostate-specific antigen; tPSA, total PSA; 1/ft PSA, the reciprocal of free-to-total PSA ratio; fPSAD, free prostate-specific antigen density; PSAD^adj^, adjusted PSAD; PSAD*weight, PSAD multiplied by weight (PSAD^adj)^.

**Figure 3:**
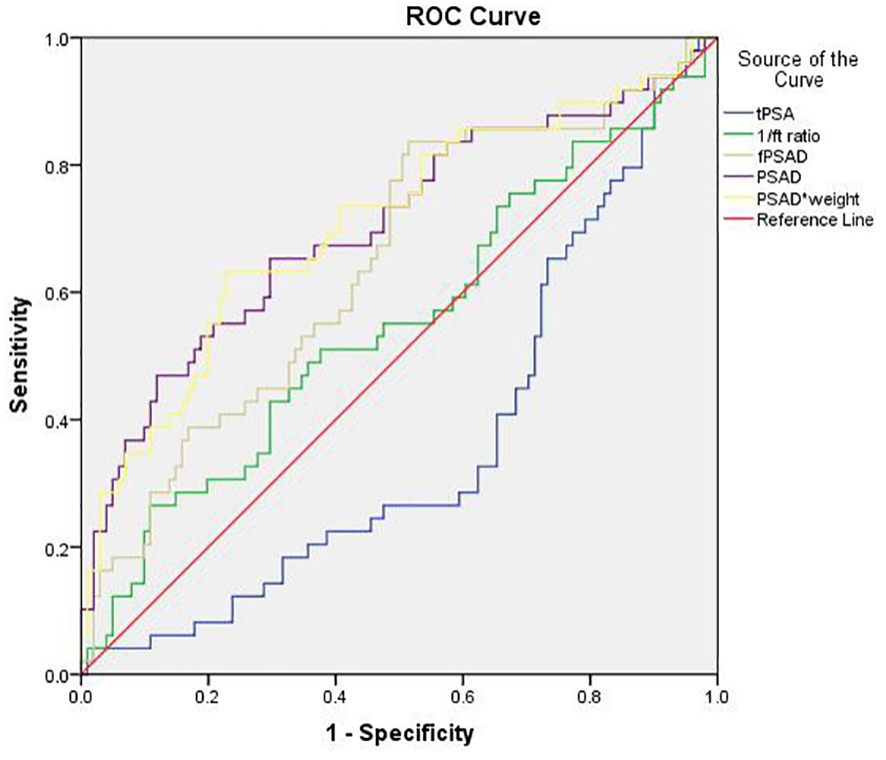
Comparison of the diagnostic efficacy of tPSA, f/t PSA, fPASD, PSAD, and PSAD^adj^ for PCa in patients with PSA levels ranging from 4.0 ng/ml to 10.0 ng/ml. PCa, prostate cancer; PSA, prostate-specific antigen; tPSA, total PSA; 1/ft PSA, the reciprocal of free-to-total PSA ratio; fPSAD, free prostate-specific antigen density; PSAD^adj^, adjusted PSAD; PSAD*weight, PSAD multiplied by weight (PSAD^adj)^.

The predictive values of tPSA, f/tPSA, fPSAD, PSAD, and PSAD^adj^ for PCa were analyzed by determining the AUC for each of the tests in all included men (**Figure 2**). The AUC values for tPSA, f/tPSA, fPSAD, PSAD, and PSAD^adj^ were 0.633, 0.656, 0.730, 0.778, and 0.780, respectively. Based on ROC analysis, a threshold of 17.804 yielded a Youden’s index maximum of 0.449, a sensitivity of 63.3%, and a specificity of 81.6% for PSAD^adj^. The Youden’s index values of tPSA, f/tPSA, fPSAD, and PSAD were 0.240, 0.308, 0.353, and 0.439, respectively, all of which were lower than that of PSAD^adj^ (**Table 3**). As the sensitivity and specificity for diagnosing PCa are limited in the PSA gray zone of 4–10 ng/ml, we further investigated the AUC of PSA-related parameters for the PCa detection of biopsied men in the gray zone (**Figure 3**). The results showed that the AUC for PSAD^adj^ remained the highest (0.709, 95% CI: 0.616–0.802), and the AUC values for tPSA, f/tPSA, fPSAD, and PSAD were 0.364, 0.551, 0.645, and 0.703, respectively. The PSAD^adj^ was superior to tPSA, f/tPSA, fPSAD, and PSAD in the diagnostic specificity for PCa, and its sensitivity was slightly lower than that of PSAD and fPSAD. The Youden’s index of PSAD^adj^ (0.405) remained the highest among all PSA-related predictors (**Table 4**). For those in the PSA gray zone, we recommended a PSAD^adj^ threshold of 10.732 with a sensitivity of 63.3% and specificity of 77.2% for the diagnosis of PCa. In summary, the specificity of PSAD^adj^ and PSAD was far superior despite their slightly poor sensitivity, especially for those in the PSA gray zone, indicating they have the highest accuracy in the diagnosis of PCa. Of note, the diagnostic efficacy of PSAD^adj^ was higher than that of PSAD for detecting PCa.

**Table 3:** AUC values of PSA-related parameters for PCa detection in the whole cohort.

Predictors	AUC	95% CI	Cutoff	Sensitivity	Specificity	Youden’s index
tPSA	0.633	0.573-0.693	12.486	56.5%	67.5%	0.240
f/tPSA	0.656	0.597-0.715	0.131	46.3%	84.5%	0.308
fPSAD	0.730	0.677-0.783	0.036	67.3%	68.0%	0.353
PSAD	0.778	0.728-0.827	0.282	59.9%	84.0%	0.439
PSAD^adj^	0.780	0.731-0.829	17.804	63.3%	81.6%	0.449

AUC, area under curve; PSA, prostate-specific antigen; PCa, prostate cancer; tPSA, total prostate-specific antigen; f/t PSA, free/total prostate specific antigen ratio; fPSAD, free prostate-specific antigen density; PSAD^adj^, adjusted PSAD.

**Table 4:** AUC values of PSA-related parameters for PCa detection in patients with PSA levels ranging from 4.0 ng/ml to 10.0 ng/ml (n=170).

Predictors	AUC	95% CI	Cutoff	Sensitivity	Specificity	Youden’s index
tPSA	0.364	0.271-0.457	6.481	28.6%	37.6%	-0.338
f/tPSA	0.551	0.450-0.653	0.168	42.9%	70.3%	0.132
fPSAD	0.645	0.551-0.740	0.022	83.7%	48.5%	0.322
PSAD	0.703	0.609-0.798	0.140	65.3%	70.3%	0.356
PSAD^adj^	0.709	0.616-0.802	10.732	63.3%	77.2%	0.405

AUC, area under curve; PSA, prostate-specific antigen; PCa, prostate cancer; tPSA, total prostate-specific antigen; f/t PSA, free/total prostate specific antigen ratio; fPSAD, free PSAD; PSAD^adj^, adjusted PSAD.

## Discussion

Our study, for the first time globally, proposed the new parameter PSAD^adj^ for the diagnosis of PCa and validated its predictive value after comparing it with other commonly used PSA-derived parameters. As this new biomarker was relatively easy to acquire, it could be directly used in clinical practice for PCa prediction, especially for those men in the PSA gray zone. However, the prediction efficacy of PSAD^adj^ needs further validation from other medical centers.

PSA is a glycoprotein enzyme secreted by prostate epithelial cells that could be released into the blood circulation when tumors or other benign lesions cause damage within the cortex, basal cell layer, or basement membrane barrier layers of prostate (15). Thus, PSA is not cancer-specific, and PCa, BPH, or prostatitis will overlap at certain PSA values, especially in the gray zone. Therefore, new indicators are needed to more accurately clinically diagnose PCa. PSAD was extensively used in clinical practice to improve the prediction efficacy of PCa. The denominator of the PSAD fraction is the prostate volume. At present, there is no standardization of prostate volume estimation; mpMRI and TRUS are the most common method used to estimate prostate volume. Lee JS, et al. and Choe S. et al. reported that MRI is more accurate than TRUS for determining the prostate volume after comparison with the measured volumes of freshly excised prostate (8, 16), and the European Association of Urology (EAU) guidelines suggests transabdominal ultrasound is discouraged because it overestimates the prostate volume (2). Based on these findings, we adopted mpMRI to assess prostate volume using the ellipsoid formula (length x height x width x π/6). The numerator of the PSAD fraction is the serum PSA concentration, which is influenced by the absolute PSA level secreted by the prostate or lesions and blood volume. Therefore, blood volume contributes much to the PSAD and could not be ignored. As body weight is positively correlated with blood volume, we proposed the parameter PSAD^adj^ by multiplying PSAD with body weight and hoped it could more accurately and objectively represent the true “PSAD”.

The overall positive biopsy rate in our study was 40.5% (166/410), which was comparable with that in the Chinese population from other centers, in which PCa was found in 44% of biopsies (17). The percentages of only 1 and 13 positive prostate biopsy cores accounted for the largest proportion, which were 13.9% (23/166) and 12.7% (21/166), respectively. The only positive core case was usually found to have wider or multiple lesion zones in the prostatectomy specimen. The 13 positive cores indicated an advanced stage of this case. In our study, the age, PSA-related parameters including tPSA, fPSAD, f/t PSA, PSAD, and PSAD^adj^, prostate volume, and positive MRI percentages in the PCa group were significantly different from those in the BPH group. Therefore, we further explored the odd ratios (ORs) of these parameters for the prediction of PCa presence using multivariate logistic analysis. From the four multivariate predicting models we built, we could see that age, tPSA, positive MRI, PSAD, and PSAD^adj^ were positive factors that were independently correlated with the presence of PCa, whereas f/t PSA and prostate volume were negative factors that were independently correlated with PCa occurrence. Our results were consistent with one Chinese population study (9), which reported that age (OR=1.06), PSAD (OR=1.10), and f/tPSA (OR=0.02) were independent predictors of PCa. The explanation for our result that the prostate volume was negatively correlated with the PCa detection rate was as follows: as prostate volume increases, the proportion of PCa tissue decreases, and thus the PCa detection rate decreases.

The AUC was significantly higher for PSAD^adj^ (0.780)and PSAD (0.778) than for the commonly used parameters tPSA (0.633) and f/tPSA (0.656) in our whole cohorts, indicating that PSAD^adj^ and PSAD were more predictive of PCa. Two Chinese population studies reported that the AUC values for PSAD were 0.744 (9) and 0.823 (18). Our data is situated between the two. In the present study, a cutoff of 0.282 for PSAD will produce 59.9% sensitivity and 84.0% specificity, and a cutoff of 17.804 for PSAD^adj^ will produce 63.3% sensitivity and 81.6% specificity. It should be noted that the previously mentioned cutoff values were determined when a Youden’s index maximum was yielded. For example, in our data, the PSAD of 0.1 had a sensitivity of 93.2% and specificity of 28.6%. By comparison, Teoh JY et al. (18) reported that a PSAD of 0.1 had a sensitivity of 97% and a specificity of 17.8%. The cutoff of our new parameter PSAD^adj^ had the optimal sensitivity and specificity among all PSA-derived parameters in the whole patients and should be considered a more precise biomarker when making biopsy decisions in population screening. The tPSA range from 4 to 10 ng/ml is generally regarded as a “gray zone” because 60%–75% of men with tPSA values in this range do not have PCa (19).

We found a significant AUC decrease in the predictive power of tPSA from 0.633 (0.573–0.693) in the whole cohort to 0.364 (0.271–0.457) in the PSA gray zone cohort. The f/tPSA ratio is considered the most widely used parameter for distinguishing between BPH and PCa in men with PSA levels from 4 to 10 ng/ml (20). We observed consistent results demonstrating that the diagnostic value of f/tPSA outperformed that of PSA (AUC 0.551 vs. 0.364) in the PSA gray zone cohort. In addition, our data showed that fPSAD had a higher diagnostic value for PCa than tPSA and f/tPSA, which was consistent with a recent study (3). Consistent with the results from the overall cohorts of our study, the AUC was still higher for PSAD^adj^ (0.709) and PSAD (0.703) than tPSA (0.364), f/tPSA (0.551), and fPSAD (0.645) in the PSA gray zone cohort. A PSAD cutoff value of 0.140 had a sensitivity of 65.3% and specificity of 70.3%, and a PSAD^adj^ cutoff of 10.732 had a sensitivity of 63.3% and specificity of 77.2%. Many scholars proposed different new cutoff values of PSAD for those men in the PSA gray zone (21–23). Lin YR et al. (21) suggested that a PSAD level of 0.15 ng/m was the optimal cutoff for Chinese patients with PSA levels ranging from 2.5 ng/ml to 10.0 ng/ml, and the sensitivity and specificity were 64.4% and 64.6%, respectively. Zheng XY et al. (22) determined the PSAD cutoff value of 0.134 ng/ml in Chinese men with PSA levels in the gray zone, with a sensitivity and specificity of 90% and 33.7%, respectively. Liu et al. (23) conducted a study on 197 men with PSA levels in the gray zone and identified 0.25 ng/ml as the optimal cutoff value for PCa. The sensitivity and specificity were 75.4% and 75.8%, respectively. The difference in the optimal cutoff values between our research and the previously mentioned studies could be explained by different population samples, prostate volume estimations, and biopsy methods in multiple hospitals by various physicians. Nevertheless, we proposed that the cutoff value selection of PSAD depends on PSAD’s diagnostic efficacy and purpose. When PSAD’s diagnostic efficacy is low, a lower cutoff should be used to increase sensitivity and decrease the amount of PCa cases that are missed. Nonetheless, it makes sense to select a higher cutoff value in cases with strong diagnostic efficacy to preserve a higher detection rate and prevent needless biopsies at the same time.

In summary, as demonstrated by the higher AUC and Youden index of PSAD^adj^, the diagnostic efficacy of PSAD^adj^ was higher than that of PSAD, not to mention other PSA-derived parameters. This result confirmed our hypothesis that the PSAD parameter is not the most reliable measure for predicting PCa occurrence. It should be noted that body weight was positively associated with the effective circulating blood volume in healthy people, and thus PSAD^adj^ could not be reasonably used in those patients with heart failure, hypoproteinemia, and other diseases that can cause an accumulation of water in the third space. The optimal cutoff of the new parameter PSAD^adj^ should be further explored and validated with larger sample sizes and in multiple medical centers. Moreover, the prostate volume may be more accurately evaluated by artificial intelligence using the calculus method and routinely written in the MRI report, which can avoid the error caused by variations in prostate shape. The ratios of epithelium to stroma in the prostate were another factor needing further research. We hope the new parameter PSAD^adj^ can be routinely used in clinical practice with a precise and accurate diagnostic value for PCa in the near future.

## Conclusion

In conclusion, this study introduced the new parameter PSAD^adj^ and validated its high predictive value for the diagnosis of PCa. There might be selection bias because our data were derived from a single center, and further exploration of the diagnostic value of PSAD^adj^ requires multicenter and large sample data.

## Data Availability

The raw data supporting the conclusions of this article will be made available by the authors, without undue reservation.

## References

[1] Bray F Laversanne M Sung H Ferlay J Siegel RL Soerjomataram I. Global cancer statistics 2022: GLOBOCAN estimates of incidence and mortality worldwide for 36 cancers in 185 countries. CA Cancer J Clin. (2024) 74(3):229–63. doi: 10.3322/caac.21834 38572751

[2] Cornford P van den Bergh RCN Briers E Van den Broeck T Brunckhorst O Darraugh J. EAU-EANM-ESTRO-ESUR-ISUP-SIOG guidelines on prostate cancer-2024 update. Part I: screening, diagnosis, and local treatment with curative intent. Eur Urol. (2024) 86(2):148–63. doi: 10.1016/j.eururo.2024.03.027 38614820

[3] Zou BZ Wen H Luo HJ Luo WC Xie QT Zhou MT. Value of serum free prostate-specific antigen density in the diagnosis of prostate cancer. Ir J Med Sci. (2023) 192(6):2681–7. doi: 10.1007/s11845-023-03448-w PMC10692254 37414978

[4] Chen Z Zhang J Jin D Wei X Qiu F Wang X. A novel clinically significant prostate cancer prediction system with multiparametric MRI and PSA: P.Z.A. score. BMC Cancer. (2023) 23(1):1138. doi: 10.1186/s12885-023-11306-2 37996859 PMC10668430

[5] Nordström T Akre O Aly M Grönberg H Eklund M. Prostate-specific antigen (PSA) density in the diagnostic algorithm of prostate cancer. Prostate Cancer Prostatic Dis. (2018) 21(1):57–63. doi: 10.1038/s41391-017-0024-7 29259293

[6] Yusim I Krenawi M Mazor E Novack V Mabjeesh NJ. The use of prostate specific antigen density to predict clinically significant prostate cancer. Sci Rep. (2020) 10(1):20015. doi: 10.1038/s41598-020-76786-9 33203873 PMC7672084

[7] Maggi M Panebianco V Mosca A Salciccia S Gentilucci A Di Pierro G. Prostate imaging reporting and data system 3 category cases at multiparametric magnetic resonance for prostate cancer: A systematic review and meta-analysis. Eur Urol Focus. (2020) 6(3):463–78. doi: 10.1016/j.euf.2019.06.014 31279677

[8] Choe S Patel HD Lanzotti N Okabe Y Rac G Shea SM. MRI vs transrectal ultrasound to estimate prostate volume and PSAD: impact on prostate cancer detection. Urology. (2023) 171:172–8. doi: 10.1016/j.urology.2022.09.007 36152871

[9] Song ZJ Qian JK Yang Y Wu HX Wang MY Jiang SY. PSA density in the diagnosis of prostate cancer in the Chinese population: results from the Chinese Prostate Cancer Consortium. Asian J Androl. (2021) 23(3):300–5. doi: 10.4103/aja.aja_61_20 PMC8152427 33208562

[10] Ehdaie B Vertosick E Spaliviero M Giallo-Uvino A Taur Y O'Sullivan M. The impact of repeat biopsies on infectious complications in men with prostate cancer on active surveillance. J Urol. (2014) 191(3):660–4. doi: 10.1016/j.juro.2013.08.088 24018237

[11] Washino S Okochi T Saito K Konishi T Hirai M Kobayashi Y. Combination of prostate imaging reporting and data system (PI-RADS) score and prostate-specific antigen (PSA) density predicts biopsy outcome in prostate biopsy naïve patients. BJU Int. (2017) 119(2):225–33. doi: 10.1111/bju.2017.119.issue-2 26935594

[12] Stevens E Truong M Bullen JA Ward RD Purysko AS Klein EA. Clinical utility of PSAD combined with PI-RADS category for the detection of clinically significant prostate cancer. Urol Oncol. (2020) 38(11):846.e9–846.e16. doi: 10.1016/j.urolonc.2020.05.024 32576527

[13] Washington SL 3rd Baskin AS Ameli N Nguyen HG Westphalen AC Shinohara K. MRI-based prostate-specific antigen density predicts gleason score upgrade in an active surveillance cohort. AJR Am J Roentgenol. (2020) 214(3):574–8. doi: 10.2214/AJR.19.21559 31913068

[14] Frisbie JW Van Besien AJ Lee A Xu L Wang S Choksi A. PSA density is complementary to prostate MP-MRI PI-RADS scoring system for risk stratification of clinically significant prostate cancer. Prostate Cancer Prostatic Dis. (2023) 26(2):347–52. doi: 10.1038/s41391-022-00549-y 35523940

[15] Balk SP Ko YJ Bubley GJ. Biology of prostate-specific antigen. J Clin Oncol. (2003) 21(2):383–91. doi: 10.1200/JCO.2003.02.083 12525533

[16] Lee JS Chung BH. Transrectal ultrasound versus magnetic resonance imaging in the estimation of prostate volume as compared with radical prostatectomy specimens. Urol Int. (2007) 78(4):323–7. doi: 10.1159/000100836 17495490

[17] Chen R Sjoberg DD Huang Y Xie L Zhou L He D. Prostate specific antigen and prostate cancer in chinese men undergoing initial prostate biopsies compared with western cohorts. J Urol. (2017) 197(1):90–6. doi: 10.1016/j.juro.2016.08.103 PMC5503119 27593477

[18] Teoh JY Yuen SK Tsu JH Wong CK Ho BS Ng AT. The performance characteristics of prostate-specific antigen and prostate-specific antigen density in Chinese men. Asian J Androl. (2017) 19(1):113–6. doi: 10.4103/1008-682X.167103 PMC5227659 26620456

[19] Roddam AW Duffy MJ Hamdy FC Ward AM Patnick J Price CP. Use of prostate-specific antigen (PSA) isoforms for the detection of prostate cancer in men with a PSA level of 2-10 ng/ml: systematic review and meta-analysis. Eur Urol. (2005) 48(3):386–99; discussion 398-9. doi: 10.1016/j.eururo.2005.04.015 15982797

[20] Catalona WJ Partin AW Slawin KM Brawer MK Flanigan RC Patel A. Use of the percentage of free prostate-specific antigen to enhance differentiation of prostate cancer from benign prostatic disease: a prospective multicenter clinical trial. JAMA. (1998) 279(19):1542–7. doi: 10.1001/jama.279.19.1542 9605898

[21] Lin YR Wei XH Uhlman M Lin XT Wu SF Diao PF. PSA density improves the rate of prostate cancer detection in Chinese men with a PSA between 2.5-10.0 ng ml (-1) and 10.1-20.0 ng ml (-1): a multicenter study. Asian J Androl. (2015) 17(3):503–7. doi: 10.4103/1008-682X.142129 PMC4430959 25475661

[22] Zheng XY Xie LP Wang YY Ding W Yang K Shen HF. The use of prostate specific antigen (PSA) density in detecting prostate cancer in Chinese men with PSA levels of 4-10 ng/mL. J Cancer Res Clin Oncol. (2008) 134(11):1207–10. doi: 10.1007/s00432-008-0400-8 PMC12161731 18446367

[23] Liu J Wang ZQ Li M Zhou MY Yu YF Zhan WW. Establishment of two new predictive models for prostate cancer to determine whether to require prostate biopsy when the PSA level is in the diagnostic gray zone (4-10 ng ml-1). Asian J Androl. (2020) 22(2):213–6. doi: 10.4103/aja.aja_46_19 PMC7155794 31169140

